# Parliament and the Profession

**Published:** 1918-04-27

**Authors:** 


### PARLIAMENT AND THE PROFESSION.

### Dispensers in the Army.

Mr. Rowlands asked the Under-Secretary of State for War a question as to qualified pharmacists in the Army whose services are not being utilised as dispensers. Mr. Macpherson said that up to the present the supply of pharmacists and dispensers for the Army has been sufficient to meet the demand, and it has not been found necessary to withdraw these men from other units. The fact that there are a number of dispensers available in corps other than the Royal Army Medical Corps has not been lost sight of.

### Venereal Diseases.

The Home Secretary, in dealing with several questions in respect of the new Defence of the Realm Regulation as to the prosecution of women knowingly communicating venereal disease to sailors or soldiers, foreshadowed that when the Criminal Law Amendment Bill is reintroduced at an early date in the House of Lords it will contain a clause, applying to persons of both sexes, to provide similar protection to the general public.

### Hospital Ship "Glenart Castle."

Dr. Macnamara informed Sir F. Hall that no report of the submarine having fired on the occupants of the boats of the *Glenart Castle* appears in the official papers dealing with inquiries held into the circumstances attending the loss of the ship.

### Medical Treatment of Discharged Irish Soldiers.

The Minister of Pensions informed Mr. Nugent that a conference was recently held at Dublin between representatives of the Pensions Ministry, the Irish Insurance Commission, the Local Government Board for Ireland, and the medical profession, at which the doctors agreed to accept a remuneration on a capitation basis of 12s. 6d. in the urban districts and 15s. in the rural districts of Ireland on a panel system,

